# Integrated Multi‐Time‐Point ^18^F‐FAPI PET/CT and Immune Histopathology for Predicting Pathological Complete Response in Microsatellite‐Stable Locally Advanced Rectal Cancer Treated With Neoadjuvant Radiotherapy, Chemotherapy, and Immunotherapy

**DOI:** 10.1002/mco2.71072

**Published:** 2026-10-13

**Authors:** Minyi He, Huiting Yang, Yunxing Shi, Rongqin Zhang, Taixuan Wan, Xiaolin Pang, Zhanwen Zhang, Liang Huang

**Affiliations:** ^1^ Department of General Surgery (Colorectal Surgery), The Sixth Affiliated Hospital Sun Yat‐sen University Guangzhou China; ^2^ Guangdong Provincial Key Laboratory of Colorectal and Pelvic Floor Diseases, The Sixth Affiliated Hospital Sun Yat‐sen University Guangzhou China; ^3^ Biomedical Innovation Center, The Sixth Affiliated Hospital Sun Yat‐sen University Guangzhou China; ^4^ Department of Nuclear Medicine, The Sixth Affiliated Hospital Sun Yat‐sen University Guangzhou China; ^5^ Department of Radiation Oncology, The Sixth Affiliated Hospital Sun Yat‐sen University Guangzhou China; ^6^ Department of General Surgery, The Sixth Affiliated Hospital Sun Yat‐sen University Guangzhou China

**Keywords:** cancer‐associated fibroblasts, FAPI PET/CT, multi‐time‐point imaging, pathological complete response, rectal cancer, tertiary lymphoid structures

## Abstract

Predicting pathological complete response (pCR) after neoadjuvant short‐course radiotherapy combined with immunotherapy and chemotherapy remains challenging in microsatellite‐stable (MSS) locally advanced rectal cancer. In this prospective exploratory study (ClinicalTrials.gov identifier: NCT06608537), 49 patients received short‐course radiotherapy followed by sintilimab plus CAPOX and subsequently underwent radical surgery. We investigated whether treatment response could be better characterized by integrating two complementary biological dimensions: baseline immune organization and treatment‐related stromal remodeling captured by multi‐time‐point ^1^ ^8^F‐FAPI PET/CT. The pCR rate was 49.0% (24/49), and serial ^1^ ^8^F‐FAPI imaging revealed distinct stromal kinetic patterns between response groups. Integration of intratumoral tertiary lymphoid structure (iTLS) count, B‐cell density, preoperative tumor‐to‐iliacus muscle ratio (TIMR) at 30 min, and the delayed TIMR retention index from 90 to 120 min (RI_34_) yielded a four‐variable model with a mean AUC of 0.874 in repeated nested cross‐validation. Complementary preclinical experiments provided functional support for a CAF–TGFβ–B‐cell/TLS axis linking stromal regulation, immune organization, and treatment resistance. These findings highlight the complementary value of spatial immune organization and dynamic FAPI‐defined stromal kinetics and provide a biologically informed framework for post‐neoadjuvant, preoperative response stratification, pending prospective multicenter validation.

## Graphical abstract

Serial multi‐time‐point ^1^ ^8^F‐FAPI PET/CT‐derived stromal kinetics and baseline immune‐histopathological features, including iTLS count and B‐cell density, jointly predicted pCR in MSS locally advanced rectal cancer. The resulting four‐variable model achieved a mean nested cross‐validated AUC of 0.874. Preclinical findings supported a plausible CAF–TGFβ–B‐cell/TLS axis through which stromal immune suppression may contribute to treatment resistance.

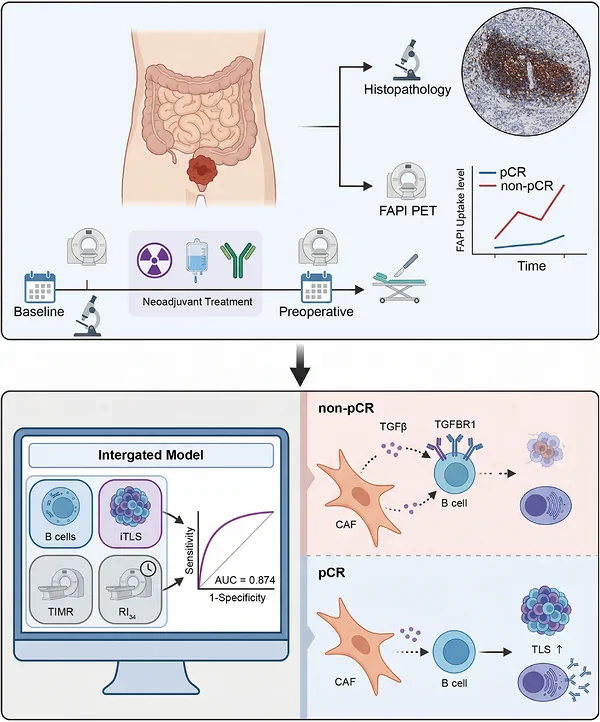

## 1 Introduction

Colorectal cancer (CRC) is the third most commonly diagnosed malignancy and the second leading cause of cancer‐related deaths globally [1, 2]. Locally advanced rectal cancer (LARC) exemplifies a major therapeutic challenge, requiring a delicate balance between achieving radical tumor control and enabling organ preservation [3]. The current standard of neoadjuvant chemoradiotherapy (nCRT) followed by surgery achieves a pathological complete response (pCR) only in a minority of patients, indicating a substantial unmet need for more effective strategies [4].

Recent studies indicate that combining short‐course radiotherapy (SCRT) with PD‐1 inhibitors and chemotherapy significantly enhances pCR rates, even in microsatellite stable (MSS) LARC [5, 6, 7, 8]. However, these intensified regimens are frequently accompanied by therapy‐induced phenomena such as pseudoprogression and pseudoresidue, which complicates the accurate assessment of treatment efficacy [9]. Consequently, conventional evaluation methods—including digital rectal exam, endoscopy, and MRI—often fail to reliably distinguish viable tumor from necrotic or fibrotic tissue, particularly following immunotherapy [9, 10].

While ^18^F‐FDG PET/CT is valuable for detecting distant metastases and recurrence in CRC by visualizing tumor glucose metabolism [11], its utility in confirming pCR is often compromised by persistent post‐treatment inflammatory uptake [12, 13, 14]. This diagnostic gap underscores the urgent need for biomarkers capable of capturing the dynamic biological changes within the tumor microenvironment during therapy.

Fibroblast activation protein (FAP), overexpressed by cancer‐associated fibroblasts (CAFs) in more than 90% of epithelial tumors, has emerged as a promising stromal target for molecular imaging [15]. FAPI PET/CT has shown diagnostic and staging value in CRC and other gastrointestinal malignancies [16, 17], and emerging studies suggest its potential for monitoring tumor immunity and therapeutic response [18, 19]. ^68^Ga‐labeled FAP inhibitor (^68^Ga‐FAPI) PET imaging has demonstrated potential for assessing tumor immunity and monitoring responses to immunotherapy in metastatic CRC [19]. In addition, ^68^Ga‐FAPI‐04 PET/MRI has shown promise in predicting pathological response in LARC [16]. Nevertheless, FAPI uptake is not cancer‐cell‐specific. FAP expression may also be induced in activated fibroblasts and myofibroblasts involved in wound healing, inflammation, fibrosis, and reparative tissue remodeling [20, 21]. While this may confound the direct interpretation of FAPI uptake as viable tumor, it also provides an opportunity to non‐invasively interrogate treatment‐related stromal activation, which represents a biologically relevant component of response after radiotherapy‐, immunotherapy‐, and chemotherapy‐containing treatment.

Notably, delayed FAPI PET imaging [22, 23, 24] has been explored to characterize time‐dependent tracer retention and improve the interpretation of active tumor‐associated stroma and reparative fibrotic or inflammatory processes. We hypothesized that multi‐time‐point ^1^ ^8^F‐FAPI PET/CT could capture dynamic stromal remodeling in LARC, offering biological information beyond pCR confirmation alone to support response assessment and patient selection for organ‐preserving strategies.

Beyond imaging, recent studies have revealed the central role of CCL19^+^ fibroblasts in the formation of tertiary lymphoid structures (TLS) [25], which are emerging biomarkers for enhanced anti‐tumor immunity and treatment efficacy in CRC [26]. Notably, TGFβ—a cytokine often over‐secreted by CAFs [27]—is a known potent suppressor of B cell activation and differentiation [28]. This raises the possibility that FAPI‐defined stromal activity and TLS‐defined immune organization represent biologically interconnected dimensions of the tumor microenvironment. Integrating dynamic FAPI‐derived stromal kinetics with baseline TLS/B‐cell immune architecture may therefore provide a more comprehensive framework for understanding and predicting treatment response than either imaging or histopathology alone.

Given the exploratory nature of the study, our primary aim was to develop an integrative response‐stratification model for pCR by combining serial multi‐time‐point ^1^ ^8^F‐FAPI PET‐derived stromal kinetic features with baseline immune contexture in MSS LARC patients. Concurrently, we sought to explore the hypothesized link between CAF activity and TLS regulation, thereby bridging clinical imaging with tumor biology and informing future personalized, function‐preserving therapeutic strategies.

## 2 Results

### 2.1 Study Cohort and Clinical Characteristics

Between December 2024 and July 2025, 52 consecutive patients with MSS LARC were initially enrolled. After applying the exclusion criteria detailed in Figure 1, 49 patients completed the full study protocol. The pCR (ypT0N0) rate was 49.0% (24/49). The cohort was predominantly high‐risk: 73.5% (36/49) of patients presented with at least one high‐risk feature, including cT4 stage (22.4%, 11/49), cN2 stage (38.8%, 19/49), MRF involvement (20.4%, 10/49), or extramural vascular invasion (46.9%, 23/49). Furthermore, a majority of tumors (79.6%, 39/49) were located within 5 cm of the anal verge. Major pretreatment demographic, tumor‐staging, and anatomical characteristics were comparable between the pCR and non‐pCR groups (Table 1). Preoperative IL‐6, a post‐treatment systemic inflammatory marker measured before surgery, was higher in the pCR group than in the non‐pCR group (*p* = 0.046).

**FIGURE 1:**
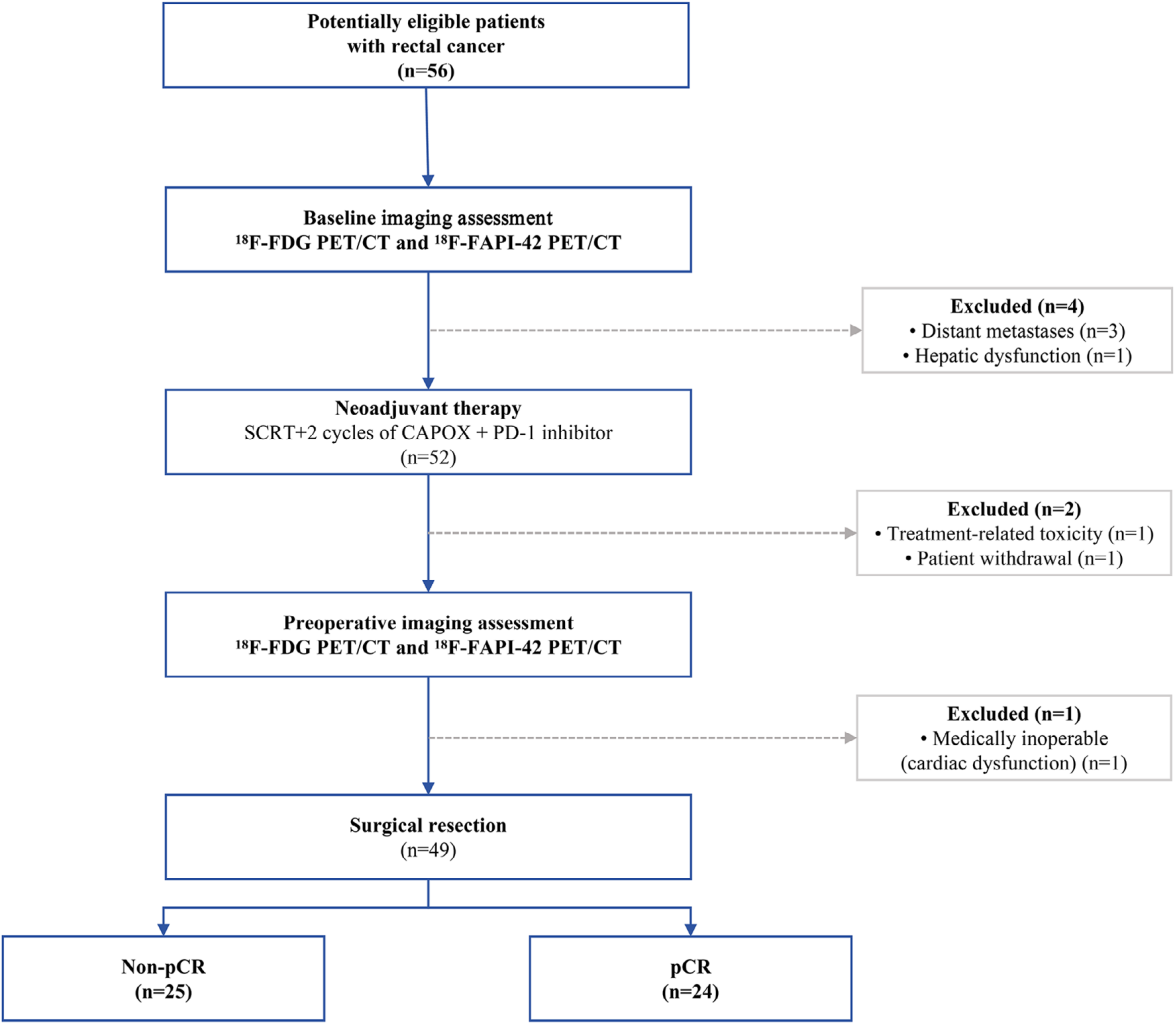
Flowchart of patient selection, treatment, and pathological response classification. Treatment response for model development was defined pathologically as pCR versus non‐pCR, rather than according to radiologic response. Accordingly, among the 49 patients included in the final analysis, 24 were classified as pCR (ypT0N0) and 25 as non‐pCR.

**TABLE 1:** Patient characteristics.

Characteristics	All patients (*N* = 49)	non‐pCR (*n* = 25)	pCR (*n* = 24)	*p*
Age (year)	56.3 (10.3)	53.8 (12.3)	58.9 (7.0)	0.081^a^
BMI (kg/m^2^)	23.1 (2.9)	23.5 (2.5)	22.6 (3.3)	0.314^a^
Gender				0.305
Male	34 (69.4%)	19 (76.0%)	15 (62.5%)	
Female	15 (30.6%)	6 (24.0%)	9 (37.5%)	
cT				0.791
T3	38 (77.6%)	19 (76.0%)	19 (79.2%)	
T4	11 (22.4%)	6 (24.0%)	5 (20.8%)	
cN				0.471
N0	8 (16.3%)	5 (20.0%)	3 (12.5%)	
N1	22 (44.9%)	9 (36.0%)	13 (54.2%)	
N2	19 (38.8%)	11 (44.0%)	8 (33.3%)	
MRF				0.725
Negative	39 (79.6%)	19 (76.0%)	20 (83.3%)	
Positive	10 (20.4%)	6 (24.0%)	4 (16.7%)	
EMVI, *n* (%)				0.674
Negative	26 (53.1%)	14 (56.0%)	12 (50.0%)	
Positive	23 (46.9%)	11 (44.0%)	12 (50.0%)	
Clinical stage				0.702
II	8 (16.3%)	5 (20.0%)	3 (12.5%)	
III	41 (83.7%)	20 (80.0%)	21 (87.5%)	
Distance from anal verge (cm)	4.2 (1.8)	4.1 (1.9)	4.3 (1.8)	0.601^a^
Baseline				
CEA (ng/mL)	7.1 (10.8)	7.1 (8.6)	7.0 (13.0)	0.74^b^
CA19‐9 (U/mL)	41.8 (126.6)	38.5 (97.9)	45.2 (153.1)	0.779^b^
AFP (ng/mL)	2.8 (3.1)	3.1 (3.6)	2.5 (2.4)	0.599^b^
Preoperative				
CEA (ng/mL)	1.9 (1.9)	1.9 (2.3)	1.9 (1.5)	0.814^b^
CA19‐9 (U/mL)	11.3 (18.4)	11.9 (21.5)	10.7 (15.4)	0.939^b^
AFP (ng/mL)	3.0 (1.7)	3.4 (0.9)	2.5 (2.2)	0.315^a^
CD4^+^ cell count (cells/µL)	395.5 (134.0)	377.9 (120.5)	418.5 (151.6)	0.286^b^
CD8^+^ cell count (cells/µL)	320.1 (156.0)	298.1 (150.2)	348.9 (164.7)	0.393^a^
CD3^+^ cell count (cells/µL)	764.3 (271.2)	722.4 (256.4)	819.1 (290.5)	0.351^a^
CD4/CD8 (ratio)	1.4 (0.7)	1.5 (0.6)	1.4 (0.7)	0.439^b^
IL‐6 (pg/mL)	4.8 (4.2)	3.7 (2.9)	6.0 (5.3)	**0.046** ^b^

*Note*: Qualitative data are presented as *n* (%), continuous data are presented as mean (SD). *p* values of qualitative data were calculated using chi‐square test or Fisher's exact test.

^a^ Student's *t*‐test for comparison between non‐pCR and pCR.

^b^ Mann–Whitney *U* test for comparison between non‐pCR and pCR.

### 2.2 Predictive Value of Serial PET Parameters

At baseline PET/CT, the ^1^ ^8^F‐FAPI TIMR differed significantly between response groups (*p* = 0.040). At preoperative PET/CT, MTV and TLG showed discriminatory value for pCR (pCR vs. non‐pCR: MTV: 1.9 ± 2.3 cm^3^ vs. 6.4 ± 7.5 cm^3^, *p* = 0.043; TLG: 7.1 ± 6.4 g/mL × cm^3^ vs. 56.8 ± 164.4 g/mL × cm^3^, *p* = 0.027). The percentage changes (Δ) from baseline to preoperative scans also differed significantly between groups, with a greater reduction in ^1^ ^8^F‐FDG TLG observed in pCR patients (pCR vs. non‐pCR: ΔTLG%: −91.9% ± 8.3% vs. −70.9% ± 59.4%, *p* = 0.020) and a more pronounced decrease in ^1^ ^8^F‐FAPI TIMR (pCR vs. non‐pCR: ΔTIMR%: −56.6% ± 18.5% vs. −41.6% ± 19.4%, *p* = 0.015) (Table 2, Table S1).

**TABLE 2:** Quantitative ^18^F‐FAPI PET parameters at baseline and preoperative, stratified by pathologic response.

Parameter	Subparameter	non‐pCR (*n* = 25)	pCR (*n* = 24)	*p*
Baseline ^18^F‐FAPI	SUVmax (g/mL)	8.0 (1.8)	8.6 (2.2)	0.284^a^
	SUVmean (g/mL)	4.4 (1.0)	4.9 (1.2)	0.174^a^
	FTV (cm^3^)	17.7 (9.4)	15.3 (10.5)	0.267^b^
	TLF (g/mL × cm^3^)	79.0 (50.3)	72.2 (43.1)	0.835^b^
	SUVpeak (g/mL)	6.3 (1.5)	7.0 (1.4)	0.105^a^
	TLR (%)	15.0 (5.0)	17.0 (5.1)	0.180^a^
	TIMR (%)	10.0 (2.5)	12.0 (3.8)	**0.040** ^a^
Preoperative ^18^F‐FAPI	SUVmax (g/mL)	4.7 (1.7)	4.2 (1.6)	0.322^a^
	SUVmean (g/mL)	3.1 (0.9)	2.8 (0.7)	0.268^a^
	FTV (cm^3^)	6.8 (7.2)	4.0 (4.2)	0.078^b^
	TLF (g/mL × cm^3^)	21.0 (22.9)	13.1 (14.2)	0.149^b^
	SUVpeak (g/mL)	4.4 (1.2)	3.8 (1.1)	0.207^a^
	TLR (%)	8.9 (4.5)	8.0 (3.9)	0.519^a^
	TIMR (%)	5.6 (1.9)	4.8 (1.9)	0.125^b^
Δ baseline to preoperative ^18^F‐FAPI^c^	ΔSUVmax%	−36.4 (33.9)	−48.3 (24.4)	0.249^b^
	ΔSUVmean%	−24.8 (30.6)	−38.0 (22.9)	0.173^b^
	ΔFTV%	−67.4 (20.5)	−58.8 (82.8)	0.248^b^
	ΔTLF%	−76.2 (16.3)	−71.9 (51.7)	0.218^b^
	ΔSUVpeak%	−22.6 (32.4)	−41.2 (19.9)	0.097^a^
	ΔTLR%	−31.1 (51.4)	−48.6 (32.8)	0.206^b^
	ΔTIMR%	−41.6 (19.4)	−56.6 (18.5)	**0.015** ^b^

*Note*: TBR = Tumor SUVmax/background SUVmean. Data are presented as mean (SD).

^a^ Welch's *t*‐test for comparison between non‐pCR and pCR.

^b^ Mann–Whitney *U* test for comparison between non‐pCR and pCR.

^c^ When calculating the change rate of ^18^F‐FAPI‐avid parameters, the preoperative FAPI parameters used for the calculation were those obtained 60 min after ^18^F‐FAPI injection.

In preoperative evaluation, dynamic ^18^F‐FAPI PET imaging kinetics provided additional predictive insight. Analysis of delayed‐phase kinetics revealed that patients achieving pCR exhibited significantly lower rates of change, as reflected in both SUVmax and the TIMR, compared to non‐pCR patients (pCR vs. non‐pCR: RI_24_^18^F‐FAPI SUVmax: 1.4% ± 13% vs. 11% ± 15%, *p* = 0.045; RI_24_^18^F‐FAPI TIMR: 13% ± 37% vs. 43% ± 37%, *p* = 0.018). Representative baseline and preoperative images are shown in Figure 2A,B; serial multi‐time‐point ^18^F‐FAPI images are displayed in Figure 2C,D. Serial kinetic analysis revealed distinct patterns: patients achieving pCR maintained relatively low and stable ^18^F‐FAPI uptake (SUVmax) over time, whereas non‐pCR patients exhibited greater fluctuation with a higher peak (Figure 2E). A similar divergence was observed in the preoperative TIMR trajectory, which increased more markedly over time in non‐responders (Figure 2F).

**FIGURE 2:**
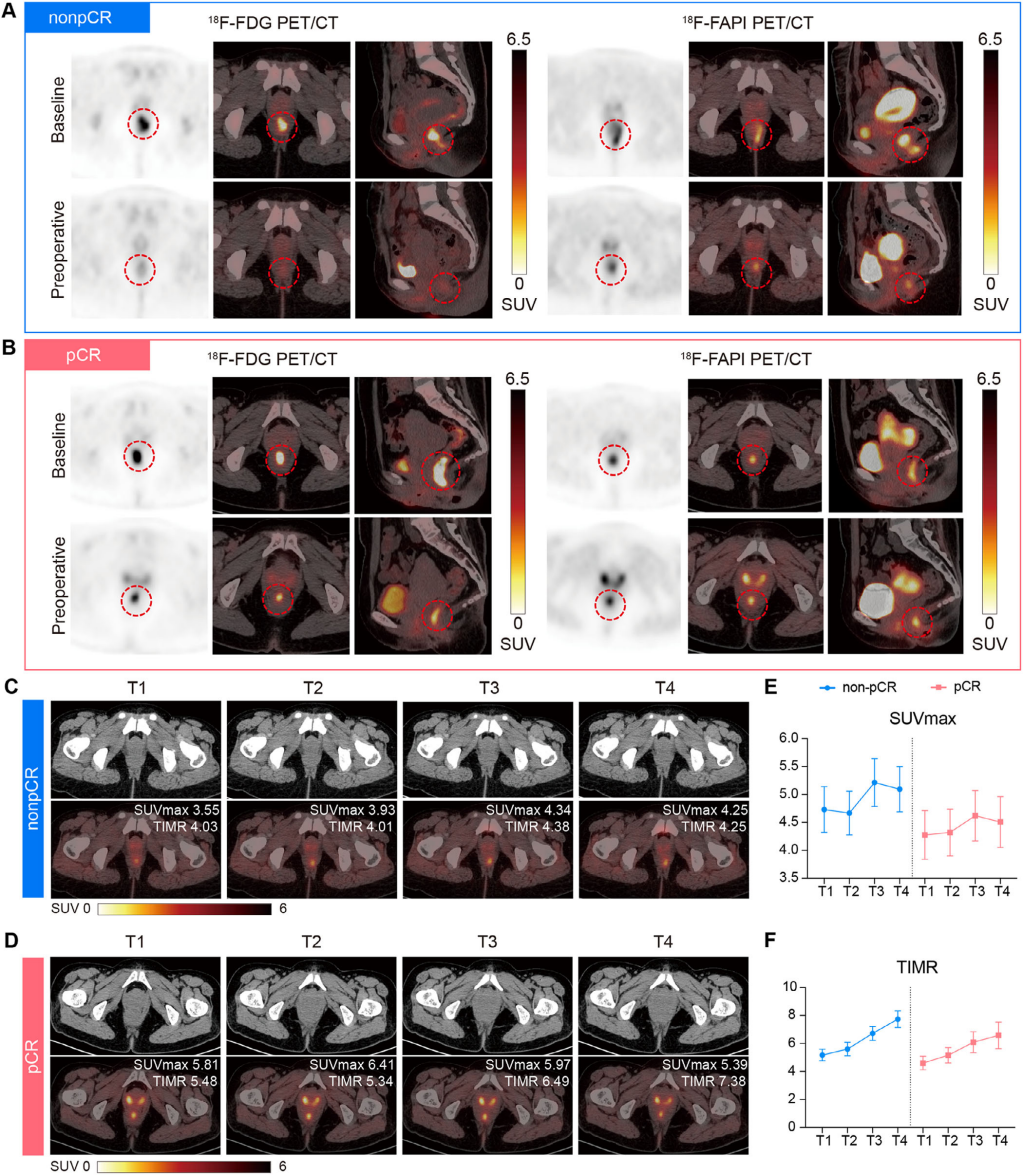
Multi‐time‐point PET imaging distinguishes treatment response. (A, B) Representative baseline and preoperative ^1^ ^8^F‐FDG and ^1^ ^8^F‐FAPI PET/CT images from a responder and a non‐responder, respectively. (C, D) Representative serial ^1^ ^8^F‐FAPI PET/CT images at four preoperative time points (*T*_1_ = 30 min, *T*_2_ = 60 min, *T*_3_ = 90 min, *T*_4_ = 120 min) in the same two patients. (E) The change of preoperative ^1^ ^8^F‐FAPI SUVmax among these four time points. (F) The change of preoperative 18F‐FAPI TIMR among these four time points.

### 2.3 Histopathological and Tumor Microenvironment Correlates of pCR

Given these distinct imaging patterns, we hypothesized that they reflected fundamental differences in the baseline tumor microenvironment. Therefore, we assessed baseline immune‐contexture architecture through histopathological and immunohistochemical analysis of baseline biopsies and evaluated its association with response. Representative histology images from patients with pCR and non‐pCR are shown in Figure 3A.

**FIGURE 3:**
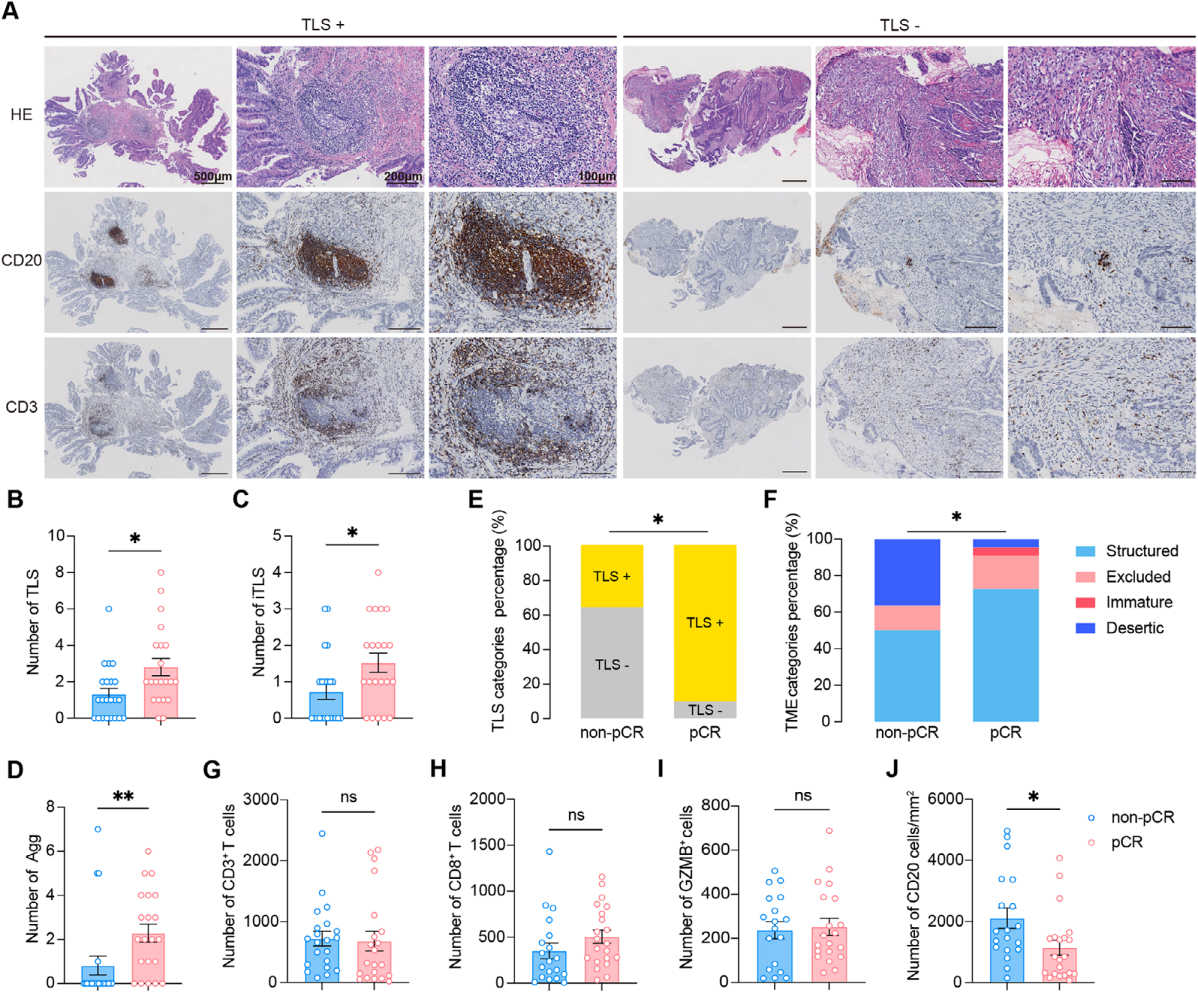
Baseline tumor microenvironment features predict pathological response. (A) Representative hematoxylin and eosin (H&E) staining and immunohistochemical (IHC) staining for CD20 and CD3 in baseline rectal cancer biopsies. Images compare regions with tertiary lymphoid structures (TLS+) from a patient who achieved pathological complete response (pCR) to regions lacking TLSs (TLS−) from a patient with non‐pCR. Scale bars: 500, 200, and 100 µm. (B–D) Quantitative analysis of TLS features in baseline tumors, stratified by ultimate pCR status. Data are presented as mean ± SD (*n* = 43). (E) Distribution of patients based on TLS status according to pCR outcome. (F) Proportional distribution of TME categories among pCR and non‐pCR patients. (G–J) Quantitative analysis of immune cell infiltration and TLS features in baseline tumors, stratified by ultimate pCR status. Data are presented as mean ± SD (*n* = 43).

The presence of TLS in baseline biopsies was significantly associated with a higher rate of pCR. This association was observed for overall TLS presence (*p* = 0.019), as well as for the intratumoral TLS (iTLS, *p* = 0.022) and aggregated (Agg, *p* = 0.001) subtypes (Figure 3B–D). Classifications based on TLS categories and the overall TME phenotype also correlated with pCR outcome (Figure 3E,F).

Notably, while the density of CD3^+^ or CD8^+^ T cells showed no association with response (Figure 3G–I), the baseline density of B cells was significantly lower in patients who achieved pCR (median: 850.4 cells/mm^2^, IQR 288.0–1462.7) than non‐responders (median: 1682.5 cells/mm^2^, IQR 1121.1–2948.7) (Figure 3J).

To explore the histopathological basis of pretreatment ^18^F‐FAPI uptake, we additionally assessed α‐SMA and FAP‐α expression in available paired biopsy specimens. Pretreatment ^18^F‐FAPI SUVmax showed modest positive correlations with α‐SMA H‐score (*r* = 0.335, *p* = 0.032) and FAP‐α H‐score (*r* = 0.359, *p* = 0.021), supporting an association between FAPI uptake and CAF‐enriched tumor stroma (Figure S1).

### 2.4 Development of an Integrative Predictive Model for pCR

Among 41 patients with available conventional restaging MRI, 20 achieved pCR. MRI showed high specificity but low sensitivity for identifying pCR (95.2% [20/21] and 10.0% [2/20], respectively; Table S5). We therefore evaluated whether PET‐derived and histopathological variables could provide complementary information for pCR prediction.

#### 2.4.1 Identification of Predictive Variables

To identify candidate predictors for model construction, we first performed univariate and receiver operating characteristic (ROC) analyses across all clinical, PET, and histopathological variables. Several features showed a strong association with pCR (Figure 4A), including the number of Aggregates (Agg; AUC = 0.76, *p* < 0.001), preoperative ^18^F‐FDG TLG (AUC = 0.73, *p* = 0.010), the number of TLS (AUC = 0.72, *p* = 0.004), the density of B cells (AUC = 0.71, *p* = 0.010), and multiple ^18^F‐FAPI parameters (e.g., RI_24_^18^F‐FAPI TIMR, AUC = 0.71, *p* = 0.020). Notably, while preoperative ^18^F‑FDG TLG achieved high sensitivity (100%) for pCR, its specificity was limited at 38.9%. In contrast, dynamic ^18^F‑FAPI parameters provided a more balanced predictive profile. Specifically, RI_24_^18^F‐FAPI TIMR (AUC = 0.71, *p* = 0.020) demonstrated a sensitivity of 81.3% and a specificity of 60.0%, while RI_34_^18^F‐FAPI TIMR (AUC = 0.71, *p* = 0.023) achieved balanced sensitivity and specificity of 75.0% (Table S2).

**FIGURE 4:**
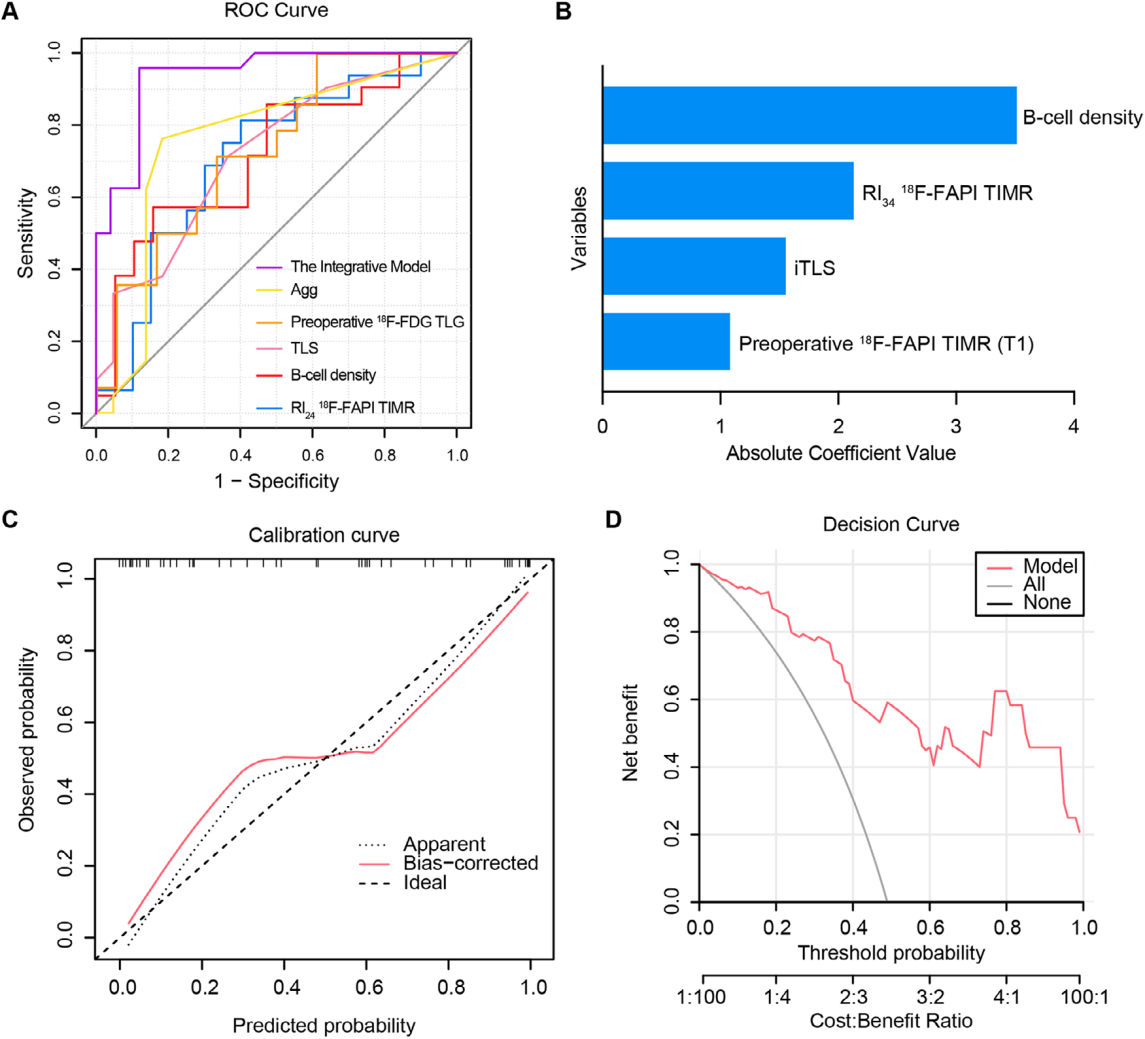
Development and internal validation of the integrative predictive model for pCR. Receiver operating characteristic (ROC) curves are shown for the five key variables identified in univariate analysis: aggregated count (Agg; AUC = 0.76), preoperative ^1^ ^8^F‐FDG TLG (AUC = 0.73), TLS count (AUC = 0.72), the density of B cells (AUC = 0.71), and RI_24_ ^1^ ^8^F‐FAPI TIMR (AUC = 0.71). The ROC curve of the final multivariate model, evaluated through repeated nested cross‑validation, is also displayed (mean AUC = 0.874, 95% CI: 0.858–0.890). (A) Absolute *β* coefficients of the final model. (B) Calibration plot of the integrative model. (C) Decision curve analysis (DCA) of the integrative model. Signed *β* coefficients, ORs, 95% CIs, and scaling information are provided in Table S3.

#### 2.4.2 Development and Validation of an Integrative Predictive Model

Given the complementary yet moderate performance of individual predictors, we developed a multivariable logistic regression model to synthesize information from imaging and immune‐histopathological features. To minimize overfitting and obtain a parsimonious feature set, LASSO regression with 10‐fold cross‐validation selected four predictors for the final model: preoperative ^18^F‐FAPI TIMR at the early time point (T1), B‐cell density, the number of iTLSs, and the retention index of ^18^F‐FAPI TIMR between delayed time points (RI_34_^18^F‐FAPI TIMR). The absolute coefficient values are shown in Figure 4B, and the signed β coefficients, ORs, 95% CIs, and scaling information are provided in Table S3.

Model performance was evaluated using repeated nested cross‐validation with 10 folds repeated 50 times. The integrative model achieved a mean AUC of 0.874 (95% CI: 0.858–0.890), with a mean sensitivity of 75.1% and a mean specificity of 74.4% (Figure 4A). Bootstrap optimism correction yielded a similar corrected AUC of 0.875 (95% CI, 0.784–0.967). The model also showed acceptable calibration and favorable net benefit on decision curve analysis (Figure 4C,D).

Leave‐one‐variable‐out analyses showed that removal of any one of the four predictors reduced the mean AUC from 0.874 to 0.760–0.801 (Table S4). These findings suggest that the performance of the full model depended on the combined predictive information provided by all four variables rather than on a single dominant predictor. In particular, exclusion of preoperative TIMR(T1) reduced the mean AUC from 0.874 to 0.766, suggesting that although TIMR(T1) was not the strongest standalone predictor, it provided incremental information within the integrated model.

As a supplementary apparent‐performance analysis, paired ROC comparison in the original dataset showed that the full model had higher discrimination than the best individual predictor, Agg (*p* = 0.008, DeLong test).

### 2.5 CAF‐Derived TGFβ Suppresses B‐Cell Function and Limits TLS Development and Organization, Contributing to Resistance to Combined Chemotherapy and Immunotherapy

Our clinical data indicate that a greater reduction in FAPI‐defined stromal activity was associated with improved treatment response. Together with the observed association between TLS and pCR, these findings prompted us to investigate whether CAF‐derived factors might modulate B‐cell function and TLS organization. We discovered that CAFs secrete higher levels of TGFβ compared to fibroblasts from normal tissues (Figure 5A). In a B cell‐CAF co‐culture system (Figure 5B), an inhibitor of TGFBR1 reversed the CAF‐mediated suppression of B cell activation, proliferation, differentiation, and IgG antibody secretion (Figure 5C–F). These findings support TGFβ as a mediator of CAF‐induced B‐cell suppression.

**FIGURE 5:**
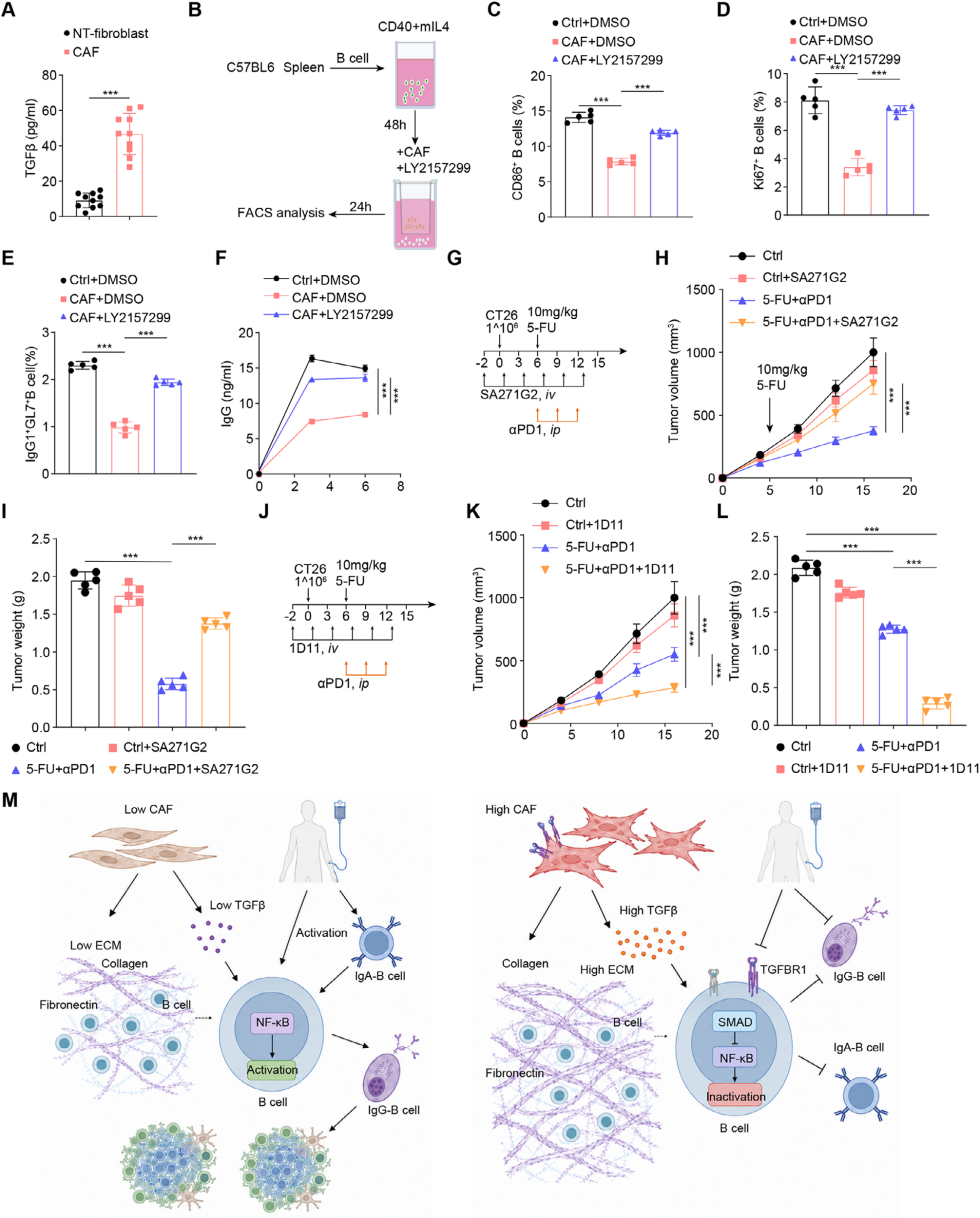
CAF‐derived TGFβ suppresses B‐cell function and limits TLS development and organization, contributing to resistance to combined chemotherapy and immunotherapy. (A) The TGFβ production was evaluated by ELISA in indicated groups in the co‐culture system (*n* = 10). (B) Schematic diagram showing the construction of the co‐culture system of primary B cells and CAF. (C) The CD86+B cells were evaluated by flow cytometry in indicated groups in the co‐culture system (*n* = 5). (D) The Ki67+B cells were evaluated by flow cytometry in indicated groups in the co‐culture system (*n* = 5). (E) The IgG1+GL7+B cells were evaluated by flow cytometry in indicated groups in the co‐culture system (*n* = 5). (F) The IgG production was evaluated by ELISA in indicated groups in the co‐culture system (*n* = 5). (G) Schematic diagram showing the construction of CT26 bearing xenografts. The mice were randomized and received 5‐FU (10 mg/kg) and SA271G2 or IgG treatment (250 µg/3 days), with anti‐PD‐1 mAb or IgG (200 µg) twice a week (*n* = 5). (H, I) Tumor growth curves (H) and tumor weight (I) for panel G. (J) Schematic diagram showing the construction of CT26 bearing xenografts. The mice were randomized and received 5‐FU (10 mg/kg) and 1D11 or IgG treatment (100 µg/3 days), with anti‐PD‐1 mAb or IgG (200 µg) twice a week (*n* = 5). (K, L) Tumor growth curves (K) and tumor weight (L) for panel J. (M) CAF‐derived TGFβ inhibits the activation, proliferation, and differentiation of B cells, thereby suppressing the formation of tertiary lymphoid structures (TLS), which in turn compromises the efficacy of combined chemotherapy and immunotherapy.

Animal experiments demonstrated that B‐cell depletion attenuated the efficacy of combined 5‐FU and anti‐PD‐1 therapy, as reflected by increased tumor volume and weight (Figure 5G–I), supporting a functional contribution of B cells to treatment response. To further validate the role of TGFβ, we employed a TGFβ‐neutralizing antibody. The results showed that TGFβ blockade significantly enhanced the therapeutic efficacy of the combined regimen (Figure 5J–L). Multiplex immunofluorescence analysis further demonstrated higher TLS counts in tumors treated with TGFβ blockade, with the highest counts observed in mice receiving 5‐FU, anti‐PD‐1, and 1D11 (Figure S2). Representative staining also showed more prominent mature TLS containing BCL6^+^ germinal centers in this group (Figure S3). Together, these findings support a role for CAF‐derived TGFβ in suppressing B‐cell function and limiting TLS abundance and organization, thereby contributing to resistance to combined chemotherapy and immunotherapy (Figure 5M).

## 3 Discussion

Accurately identifying pCR after immunotherapy‐containing neoadjuvant therapy remains challenging in MSS LARC. In this prospective exploratory cohort, we found that treatment response was associated with two complementary biological features: baseline immune organization, particularly iTLS, and treatment‐related FAP‐positive stromal remodeling captured by serial multi‐time‐point ^18^F‐FAPI PET/CT. By integrating these immune‐histopathological and imaging‐derived stromal features, the final four‐variable model achieved a mean internally validated AUC of 0.874 for pCR prediction.

The pCR rate in this single‐arm cohort was 49.0%, toward the upper range of reported outcomes for contemporary immunotherapy‐containing neoadjuvant regimens in pMMR/MSS LARC, consistent with emerging evidence that a subset of these tumors may be sensitized to PD‐1 blockade when incorporated into radiotherapy‐ and chemotherapy‐containing regimens [5, 7, 8, 29, 30]. The contribution of sintilimab itself, however, cannot be separated from that of the combined regimen without a randomized comparator. These data therefore should not be taken as evidence for routine immunotherapy intensification across all patients; instead, they point to biological heterogeneity in treatment response within MSS disease.

Conventional restaging MRI further underscored the need for complementary response biomarkers [9, 31, 32]. In the present cohort, expert‐interpreted restaging MRI showed high specificity but identified only 2 of 20 patients who ultimately achieved pCR, corresponding to a sensitivity of 10.0%. MRI remains indispensable for local staging, surgical planning, and routine response assessment, but fibrosis, inflammation, and treatment‐related tissue remodeling can obscure the distinction between viable tumor and post‐treatment change. MRI‐based radiomics studies have reported promising performance [33, 34, 35], but the present study neither developed an MRI‐radiomics model nor performed a head‐to‐head comparison with such approaches. Our results therefore position ^18^F‐FAPI PET/CT as an additional source of biological information, particularly regarding FAP‐positive stromal activity and remodeling, rather than as a replacement for MRI [16, 22].

Both ^18^F‐FDG and ^18^F‐FAPI PET were included to characterize different aspects of treatment response. ^18^F‐FDG MTV and TLG were associated with pCR, consistent with previous studies [16, 36]. However, residual low‐level FDG uptake was frequently observed at the primary tumor site even in patients who achieved pCR, possibly reflecting treatment‐related inflammation or tissue repair rather than viable residual tumor [12, 13, 14]. This inflammatory or reparative uptake may complicate post‐treatment tumor delineation and limit the ability of ^18^F‐FDG PET to distinguish residual tumor from treatment‐related changes. ^18^F‐FAPI PET interrogates a different biological compartment by reflecting FAP‐positive stromal activity and remodeling rather than glucose metabolism [17, 18, 37].

To extend this stromal assessment beyond static uptake, we used a multi‐time‐point FAPI protocol to characterize tracer kinetics [22, 23, 24]. In our cohort, patients achieving pCR showed lower and relatively stable SUVmax and a less pronounced delayed increase in TIMR, whereas non‐pCR patients exhibited greater delayed retention. The early decline followed by stabilization of FAPI uptake in pCR tumors may reflect a marked reduction in FAP‐positive activated stromal activity after effective treatment. Residual low and stable uptake could arise from wound healing, extracellular matrix remodeling, or early fibrosis within the treated tumor bed rather than persistent CAF‐rich tumor‐associated stroma [21, 38, 39]. In contrast, the gradual delayed‐phase increase observed in non‐pCR tumors may reflect persistent or regenerating FAP‐positive tumor stroma, ongoing CAF activation, and continued extracellular matrix remodeling [40]. Consistently, the final LASSO‐selected model retained both an early post‐treatment preoperative uptake parameter, TIMR(T1), and a delayed kinetic parameter, RI_34_^18^F‐FAPI TIMR. These findings suggest that TIMR(T1) reflects the magnitude of residual FAP‐positive stromal activity, whereas RI_34_ may capture whether this activity behaves as resolving treatment‐related remodeling or persistent tumor‐associated stromal activation.

Baseline immune architecture added another dimension to these imaging findings. Baseline iTLS count was positively associated with pCR, whereas CD8^+^ T‐cell density alone was not. This lack of association may partly reflect the relatively limited CD8^+^ T‐cell infiltration commonly observed in MSS rectal cancer, as well as the inability of cell density alone to capture spatial distribution, activation state, or functional competence. These findings suggest that organized immune architecture may be more informative than simple immune‐cell abundance in this setting. The inverse association between overall B‐cell density and pCR requires a similarly nuanced interpretation. It does not imply that B cells are uniformly detrimental, because B‐cell abundance, functional phenotype, and spatial organization are biologically distinct. Organized B‐cell immunity within iTLS may be more relevant to effective antitumor responses. Conversely, the higher overall B‐cell density in non‐responders may reflect different functional states, including enrichment of regulatory B‐cell subsets [41, 42]. Because the clinical IHC quantified overall B‐cell density without subset‐specific phenotyping, this explanation remains unproven.

To translate these complementary imaging and immune‐histopathological signals into a predictive framework, we developed a four‐variable model integrating preoperative FAPI uptake and kinetics with B‐cell density and iTLS count. Its internally validated performance, together with the decline in discrimination after individual predictors were removed, indicates that no single biomarker accounted for the model's predictive ability.

Building on the clinical associations between stromal remodeling, immune organization, and treatment response [19, 25, 27, 28], our preclinical experiments support a plausible CAF–TGFβ–B‐cell/TLS axis. CAF‐conditioned medium suppressed B‐cell activation, proliferation, differentiation, and IgG secretion in vitro, and these effects were reversed by TGFBR1 inhibition, supporting TGFβ as a mediator of CAF‐induced B‐cell suppression. In vivo, B‐cell depletion attenuated the efficacy of 5‐FU plus anti‐PD‐1 therapy, whereas TGFβ blockade enhanced treatment efficacy and was accompanied by more prominent spatial organization of T‐ and B‐cell aggregates in endpoint CT26 tumors. Together, these findings suggest that CAF‐derived TGFβ may restrain B‐cell function and limit TLS organization, thereby contributing to treatment resistance. However, the preclinical findings do not establish that the higher overall B‐cell density observed in non‐responders was driven by CAF‐derived TGFβ. Because both CAFs and B cells are functionally heterogeneous and were not analyzed at the subset level, the specific cell populations involved remain to be defined.

From a clinical perspective, the present model should be viewed as an exploratory post‐treatment response‐stratification tool rather than as a standalone basis for treatment selection. Because all patients in this cohort underwent radical surgery after neoadjuvant therapy, our data do not support omitting surgery on the basis of model prediction alone. Patients with a low predicted probability of pCR would generally remain candidates for timely total mesorectal excision, with subsequent management guided by pathological findings and current clinical standards. Those with a high predicted probability may warrant intensified multimodal reassessment using MRI, endoscopy, clinical examination, and pathological confirmation when feasible; after prospective validation, the model may help identify candidates for carefully monitored organ‐preservation protocols. For patients with TLS‐poor or stromal‐dominant response phenotypes, the present findings may also inform future biomarker‐guided trials evaluating immune‐priming or stromal‐directed strategies, although these approaches remain investigational.

This study has several limitations. First, the single‐center exploratory cohort was small, and all validation was internal; prospective multicenter validation remains essential. Second, dynamic ^18^F‐FAPI parameters were unavailable in 9 patients and were imputed for full‐cohort modeling, requiring confirmation in cohorts with complete imaging. Third, the comparator was conventional restaging MRI rather than MRI‐radiomics, precluding direct comparison with published radiomics models. Fourth, the cellular source of post‐treatment FAPI uptake cannot be resolved without spatially matched post‐treatment histology. Fifth, the CT26 experiments provided mechanistic support but did not reproduce the human regimen: CT26 is a relatively immunogenic pMMR/MSS‐like subcutaneous model [43], and neither radiotherapy nor serial FAPI micro‐PET/CT was included. Radiotherapy‐containing models integrating serial FAPI imaging with spatial stromal–immune profiling are therefore needed to further evaluate the proposed imaging–stromal–immune relationship.

In conclusion, baseline spatial immune organization, particularly iTLS, and serial ^18^F‐FAPI PET‐derived stromal kinetics jointly informed pCR prediction in this exploratory MSS LARC cohort. Their integration enabled post‐treatment response stratification beyond either feature alone. Prospective validation will be necessary before the model can be considered for use in clinical decision‐making or organ‐preservation pathways.

## 4 Materials and Methods

### 4.1 Patients

This prospective single‐center study enrolled newly diagnosed patients with LARC. Eligible participants were aged 18–75 years, with histologically confirmed rectal adenocarcinoma classified as MSS and as clinical stage T3‐4/N^+^M0 based on magnetic resonance imaging (MRI) and PET according to the criteria set forth by the American Joint Committee on Cancer (AJCC) eighth edition. Patients were required to have an Eastern Cooperative Oncology Group (ECOG) performance status of 0 or 1, a tumor inferior margin located less than 10 cm above the anal verge, and adequate organ function. Patients with a history of prior antitumor treatment or those with known or suspected allergies to any drug used in the study were excluded.

This study was conducted in accordance with the Declaration of Helsinki and the International Council for Harmonisation Good Clinical Practice (ICH‐GCP) guidelines. It was registered at ClinicalTrials.gov (identifier: NCT06608537) and received approval from the local ethics committee at the Sixth Affiliated Hospital of Sun Yat‐sen University (identifier: 2025ZSLYEC‐118). All patients provided written informed consent before enrollment.

### 4.2 Sample Size

Based on an anticipated AUC improvement from 0.60 to 0.85, an assumed pCR rate of 40%, a two‐sided α of 0.05, and 80% power, 38 patients were required using PASS 15.0.5; allowing for approximately 10% attrition, the planned sample size was 43.

### 4.3 Procedures

After enrollment, baseline ^18^F‐FAPI and ^18^F‐FDG PET/CT were performed within an interval of less than 1 week for each patient for disease assessment (Figure 1). Then, the patients received neoadjuvant treatment consisting of SCRT (a total of 25 Gy in 5 days) followed by Sintilimab (200 mg intravenous drip on day 1; every 3‐week cycle for two cycles) combined with CAPOX (oxaliplatin 130 mg/m^2^ intravenous infusion over 2 h on Day 1, capecitabine 1000 mg/m^2^ orally twice daily from Day 1–14, in every 3‐week cycle for two cycles) 1 week later. Eight weeks after SCRT, the 2 imaging modalities were repeated for preoperative evaluation, followed by radical surgery within 1 week. After evaluation, all patients underwent radical surgery. pCR was defined as the absence of any remaining viable cancer cells in the resected primary tumor specimen and all sampled regional lymph nodes (ypT0N0).

### 4.4 PET/CT Imaging and MRI Imaging

For baseline or preoperative assessment, ^18^F‐FDG and ^18^F‐FAPI PET/CT were performed on two different days within 1 week. All patients fasted for at least 6 h prior to the ^18^F‐FDG PET/CT scan, and blood glucose levels were maintained within normal limits (≤ 11.1 mmol/L). For the ^18^F‐FAPI PET/CT examination, fasting was not required. Following intravenous administration of ^18^F‐FDG (3.7‐5.5 MBq/kg) or ^18^F‐FAPI‐42 (3.7‐5.5 MBq/kg), routine imaging for both tracers was initiated 60 min post‐injection using a PET/CT scanner (United Imaging, uMI780). A modified protocol was applied for preoperative ^18^F‐FAPI scans, wherein data acquisition began at 30, 60, 90, and 120 min post‐injection, yielding a total of four sequential scans. The scan range for the ^18^F‐FDG PET/CT examination extended from the skull base to the mid‐femur, while the ^18^F‐FAPI scan extended from the top of the head to the mid‐femur. A CT scan was performed first, immediately followed by 3D PET acquisition (5–6 bed positions, 2 min/bed). PET data were reconstructed iteratively (2 iterations and 20 subsets) with CT data for attenuation correction, and the PET/CT images were then co‐registered and displayed using the uWS‐MI Workstation (United Imaging Healthcare, Shanghai, China).

The pelvic MRI protocols included T1‐weighted, T2‐weighted, and diffusion‐weighted imaging (DWI). Contrast‐enhanced MRI was performed using a modified Dixon sequence on a 1.5‐T MRI unit (GE).

### 4.5 Image Interpretation

PET images were analyzed using the uWS‐MI workstation. Tumor contours were generated using a semi‐automatic segmentation approach and subsequently reviewed and manually adjusted by two experienced nuclear medicine physicians to exclude areas of physiological and clearly non‐tumoral inflammatory uptake outside the residual tumor bed. To assess background activity, spherical volumes of interest were placed entirely within the right liver lobe (2.0 cm diameter) and the iliacus muscle (1.0 cm diameter).

Standardized uptake values (SUVs) were standardized by lean body mass. For ^18^F‐FDG PET, quantitative parameters included SUVmax, SUVmean, SUVpeak, metabolic tumor volume (MTV) and total lesion glycolysis (TLG). For ^18^F‐FAPI PET, the parameters included SUVmax, SUVmean, SUVpeak, ^18^F‐FAPI‐avid tumor volume (FTV), and total lesion FAP expression (TLF). In addition, the tumor‐to‐liver ratio (TLR) and tumor‐to‐iliacus muscle ratio (TIMR) were calculated as the ratio of tumor SUVmax to background SUVmean, with background regions of interest placed in the right hepatic lobe and the iliacus muscle, respectively. The percentage change (△%) between baseline and preoperative scans for each parameter was calculated using the following formula: △% = [(preoperative value − baseline value)/baseline value] × 100%.

To comprehensively analyze tracer kinetics across all acquisition time points in preoperative ^18^F‐FAPI PET/CT, we calculated a series of retention indices (RIs). These indices were denoted as RI*_ij_*, where *i* and *j* represent different post‐injection time points (*T*_1_ = 30 min, *T*_2_ = 60 min, *T*_3_ = 90 min, *T*_4_ = 120 min). The general formula was: RI*_ij_* = [(*T_j_* _value − T*_i_* _value)/*T_i_* _value] × 100%. Specific indices of interest, such as RI_24​_ (change from *T*_2_ to *T*_4_), were derived accordingly for subsequent analysis.

Baseline pelvic MRI was used for initial clinical staging, whereas restaging MRI after neoadjuvant therapy was used for preoperative response assessment. Restaging MRI was reviewed by experienced radiologists before the final pathological results were available. Radiologic complete response was determined using a comprehensive assessment of high‐resolution T2‐weighted imaging (T2WI), DWI, apparent diffusion coefficient (ADC) maps, and nodal status. The radiologic diagnosis of cCR mandated the fulfillment of all following criteria: (a) Morphological normalization at the primary tumor site, defined by the absence of any residual mass or nodular wall thickening. (b) Characteristic post‐treatment signal patterns: homogeneous T2 hypointensity without any intermediate signal component, coupled with the absence of focal restricted diffusion (i.e., no focal high signal on high *b*‐value DWI [800–1000 s/mm^2^] with a corresponding low signal on ADC maps). (c) Absence of suspicious lymph nodes, allowing only for benign‐appearing subcentimeter nodes with a short‐axis diameter of < 5 mm.

### 4.6 Histopathological, Immunohistochemical, and Digital Pathology Analysis

Baseline biopsy samples were fixed in formalin, embedded in formalin‐fixed, paraffin‐embedded (FFPE), and sectioned into 4‐µm sections. Sections were stained with hematoxylin and eosin (H&E) or processed for immunohistochemistry (IHC) using antibodies against CD3 (Zhongshan Golden Bridge, ZA‐0503), CD8 (Zhongshan Golden Bridge, ZA‐0508), CD20 (Abcam, ab64088), and GZMB (Abcam, ab255598). Whole‐slide images were acquired using a high‐resolution scanner. Immune cell densities (cells/mm^2^) for CD3^+^, CD8^+^, CD20^+^, and GZMB^+^ cells were quantified using ImageJ. TLS were identified on H&E‐stained sections and confirmed by CD3/CD20 IHC. TLS were categorized as mature (with germinal center) or aggregate. Based on TLS spatial distribution and maturity, the tumor immune microenvironment was classified into four phenotypes as previously described: immune‐structured, with iTLSs; immune‐excluded, with mature peritumoral TLSs restricted to the tumor margin; immune‐dispersed, with scattered TILs and/or immature peritumoral lymphoid aggregates but no iTLSs; and immune‐desert, with sparse TILs and no TLSs. immune‐structured and immune‐excluded phenotypes were categorized as TLS^+^, whereas immune‐dispersed and immune‐desert phenotypes were categorized as TLS^−^.

To provide histopathological support for the stromal basis of ^18^F‐FAPI uptake, additional IHC staining for CAF‐related markers was performed on available pretreatment biopsy specimens using antibodies against α‐SMA (Abcam, ab124964) and FAP‐α (Proteintech, 27596‐1‐AP). Staining intensity and the percentage of positive stromal cells were quantified using the *H*‐score method. The *H*‐score was calculated as follows: *H*‐score = 1 × percentage of weakly stained stromal cells + 2 × percentage of moderately stained stromal cells + 3 × percentage of strongly stained stromal cells, yielding a total score ranging from 0 to 300. Quantification was performed by investigators blinded to PET parameters and pathological response. Correlations between pretreatment ^18^F‐FAPI SUVmax and α‐SMA or FAP‐α H‐scores were assessed using correlation analysis.

### 4.7 Source and Isolation of Fibroblasts

Normal tissue‐derived fibroblasts (NFs) and CAFs were isolated from paired adjacent normal colorectal tissues and primary CRC tissues, respectively, obtained from patients who underwent surgical resection at the Sixth Affiliated Hospital of Sun Yat‐sen University (Guangzhou, China). All tissue samples were collected with written informed consent, and the study was approved by the Institutional Ethics Committee of the Sixth Affiliated Hospital of Sun Yat‐sen University. Fresh surgical specimens were washed three times with PBS containing 1% penicillin‐streptomycin, minced into approximately 1 mm^3^ pieces, and digested with 0.5 mg/mL collagenase IV (Sigma‐Aldrich) and 0.2 mg/mL DNase I (Roche) in DMEM/F12 at 37°C for 1–2 h with intermittent shaking. The digested tissue suspension was filtered through a 70 µm cell strainer, centrifuged at 300 × *g* for 5 min, and the cell pellet was resuspended in fibroblast culture medium (DMEM/F12 supplemented with 10% fetal bovine serum, 100 U/mL penicillin, and 100 µg/mL streptomycin). Fibroblasts were enriched by differential adhesion: cells were plated in culture flasks and incubated for 30–40 min; non‐adherent cells were removed by gentle washing, while adherent fibroblast‐like cells were maintained in culture. The culture medium was changed every 2–3 days, and cells were passaged upon reaching 80%–90% confluence using 0.25% trypsin‐EDTA.

Mouse CAFs were isolated from subcutaneous xenograft tumors established by CT26 cells into the flanks of 6‑week‑old female BALB/c mice. When tumors reached ∼500 mm^3^, mice were euthanized and tumors were excised under sterile conditions. The tumor tissues were minced and digested using the same enzyme cocktail (collagenase IV + DNase I) as described above, followed by filtration and differential adhesion.

### 4.8 Cell Culture and In Vitro Functional Assays

CT26 murine colon carcinoma cells were obtained from Guangzhou Jennio Biotechnology with a short tandem repeat authentication certificate and were certified by the supplier to be mycoplasma‐free. All fibroblast cultures were maintained at 37°C in a humidified atmosphere containing 5% CO_2_. For all experiments, NFs and CAFs at passages 3–6 were used to minimize phenotypic drift that may occur with extended passaging. Cells were routinely monitored for mycoplasma contamination using a Mycoplasma Detection Kit (Beyotime) and were confirmed negative. Conditioned medium (CAF‐CM) was collected, and TGF‐β concentration was quantified via ELISA. Primary murine splenic B cells were isolated from C57BL/6 mice (8–12 weeks old) using CD19/B220 microbeads (Miltenyi Biotec). Naïve B cells were activated with anti‐CD40 (1 µg/mL) and IL‐4 (10–20 ng/mL) for 48 h. To test CAF‐mediated suppression, activated B cells were treated with CAF‐CM (1:1 v/v) in the presence or absence of the TGF‐β receptor I inhibitor LY2157299 (10 µM). After 24 h, cells were analyzed by flow cytometry for markers of activation (CD86), proliferation (Ki67), and differentiation (GL7, IgG1). Secreted IgG was measured by ELISA.

### 4.9 Mouse Tumor Models and In Vivo Studies

All animal experiments were reviewed and approved by the Experimental Animal Ethics Committee of Ruiye Model Animal (Guangzhou) Biotechnology Co. Ltd. (approval no. RYEth‐202505011258) and were conducted in accordance with institutional guidelines and the ARRIVE guidelines. CT26 colon carcinoma cells, a BALB/c‐derived murine colon carcinoma cell line commonly used as a syngeneic pMMR/MSS‐like CRC model [44], were inoculated subcutaneously into BALB/c mice. When tumors reached ∼50–100 mm^3^, mice were randomized into treatment groups (*n* = 5). In the B‐cell‐depletion experiment, mice received control treatment, SA271G2 alone, 5‐FU plus anti‐PD‐1, or 5‐FU plus anti‐PD‐1 plus SA271G2. In the TGFβ‐blockade experiment, mice received control treatment, 1D11 alone, 5‐FU plus anti‐PD‐1, or 5‐FU plus anti‐PD‐1 plus 1D11. 5‐FU (10 mg/kg, i.p.) was administered once on Day 6; anti‐PD‐1 (200 µg, i.p.) was administered on Days 6, 9, and 12; and SA271G2 (250 µg, i.v.) or 1D11 (100 µg, i.v.) was administered on Days −2, 0, 3, 6, 9, and 12. Tumor dimensions and body weight were monitored regularly. Tumors were harvested at the endpoint for analysis.

### 4.10 Multiplex Immunofluorescence Analysis of Murine Tumors

FFPE CT26 tumor sections were subjected to multiplex immunofluorescence staining using antibodies against CD3 (Proteintech, 17617‐1‐AP), CD4 (Proteintech, 67786‐1‐Ig), CD79A (Proteintech, 31887‐1‐AP), and CD138 (Proteintech, 10593‐1‐AP), with DAPI used for nuclear counterstaining. Images were acquired under identical conditions across treatment groups. TLS were defined as organized lymphoid aggregates containing spatially coordinated T‐cell and B‐cell components. The entire tumor section was systematically examined, and the total number of TLS per section was recorded. For CD79A^+^ cell quantification, five randomly selected non‐overlapping intratumoral fields were analyzed for each tumor, and the mean number of positive cells per field was calculated.

### 4.11 Statistical Analysis

Continuous variables are presented as mean ± standard deviation (SD), and categorical variables as number (%). Normality was assessed using the Shapiro–Wilk test. Group comparisons were performed using the Student's *t*‐test or Mann–Whitney *U* test for continuous data, and the *χ* ^2^ or Fisher's exact test for categorical data, as appropriate. For in vitro and animal experiments, comparisons among three or more groups were performed using one‐way ANOVA followed by Tukey's multiple‐comparison test. Longitudinal tumor‐growth curves were analyzed using two‐way repeated‐measures ANOVA with multiplicity‐adjusted post hoc comparisons. Correlations were assessed using Pearson's or Spearman's coefficients. The predictive performance of individual variables was evaluated by ROC curve analysis.

To develop an integrative predictive model, continuous candidate predictors were standardized before feature selection. Feature selection was performed using the least absolute shrinkage and selection operator (LASSO) regression with 10‑fold cross‑validation. After feature selection, the final multivariable logistic regression model was refitted using the selected predictors on their original or prespecified clinically interpretable scales to facilitate interpretation of the regression coefficients and odds ratios. B‐cell density was rescaled as B‐cell density/100 before model fitting; therefore, its OR represents the change in odds of pCR per 100 cells/mm^2^ increase. Model performance was evaluated using repeated nested cross‐validation with 10 outer folds, repeated 50 times. Within each outer training set, missing‐value imputation, predictor standardization, inner cross‐validation for LASSO tuning and feature selection, and logistic model fitting were performed using the training data only. The resulting preprocessing steps and fitted model were then applied to the held‐out outer fold, which was used exclusively for performance evaluation. Out‐of‐fold predicted probabilities were combined within each repeat to estimate the AUC, sensitivity, and specificity. To estimate optimism‐corrected discrimination, bootstrap validation with 500 resamples was performed. In each resample, the model was refitted and evaluated in both the bootstrap sample and the original dataset, and the average optimism was subtracted from the apparent AUC to obtain the optimism‐corrected AUC. Pairwise comparisons between the integrative model and individual predictors were performed using the DeLong test based on paired ROC curves. A two‐sided *p* value < 0.05 was considered statistically significant.

For dynamic ^18^F‐FAPI PET parameters available in 40 patients, missing values in the remaining 9 patients were addressed using multiple imputation to enable full‐cohort exploratory modeling. Model performance was interpreted in conjunction with repeated nested cross‐validation and bootstrap optimism correction. All analyses were two‑sided, with a *p*  < 0.05 considered significant. Statistical analyses were performed using R (version 4.3.0) and GraphPad Prism (version 10.0).

## Author Contributions

M.Y.H., H.T.Y., and S.Y.X. designed and directed the study. R.Q.Z., T.X.W., and X.L.P. analyzed the data, prepared the figures, and wrote the manuscript. M.Y.H., H.T.Y., S.Y.X., and X.L.P. performed all the experiments. L.H., R.Q.Z., and Z.W.Z. supervised the manuscript. L.H., Z.W.Z., and R.Q.Z. provided funding for the studies. All authors have read and approved the final manuscript.

## Funding

This work was supported by: Guangdong Province Basic and Applied Basic Research Foundation (Grant Numbers: 2023A1515220170, 2024A1515220132); Guangzhou Science and Technology Plan Project (Grant Number: 202201011199); The “1010” Project of The Sixth Affiliated Hospital, Sun Yat‐sen University (Grant Number: 1010CG (2023)‐07); The Natural Science Foundation of Guangdong Province, China (No.2023A1515011300); Medical Scientific Research Foundation of Guangdong Province of China (No. B2025186). The funders had no role in study design, data collection and analysis, decision to publish, or preparation of the manuscript.

## Ethics Statement

This prospective study was reviewed and approved by the Ethics Committee of The Sixth Affiliated Hospital, Sun Yat‐sen University (approval number: 2025ZSLYEC‐118) and was conducted in accordance with the Declaration of Helsinki. The study was registered at ClinicalTrials.gov (NCT06608537). All animal experiments were reviewed and approved by the Experimental Animal Ethics Committee of Ruiye Model Animal (Guangzhou) Biotechnology Co., Ltd. (approval no. RYEth‐202505011258). Written informed consent was obtained from all individual participants prior to their enrollment.

## Conflicts of Interest

The authors declare no conflicts of interest.

## Supporting information




**Supporting File**: mco271072‐sup‐0001‐SuppMat.docx.

## Data Availability

The datasets generated and analyzed during this prospective study are available from the corresponding author upon reasonable request.
