# Supplementary material for: Integrated Multi‐Time‐Point 18F‐FAPI PET/CT and Immune Histopathology for Predicting Pathological Complete Response in Microsatellite‐Stable Locally Advanced Rectal Cancer Treated With Neoadjuvant Radiotherapy, Chemotherapy, and Immunotherapy
Source: MedComm (2020). 2026 Oct 13;7(10):e71072. doi: 10.1002/mco2.71072 (PMC13660711; doi:10.1002/mco2.71072)
Supplement: Supplementary file 1 [file MCO2-7-e71072-s001.docx]

| **Integrated Multi-Time-Point ^18^F-FAPI PET/CT and Immune Histopathology for Predicting Pathological Complete Response in Microsatellite-Stable Locally Advanced Rectal Cancer Treated With Neoadjuvant Radiotherapy, Chemotherapy, and Immunotherapy**  *Minyi He^1,2,3#^, Huiting Yang^4#^, Yunxing Shi^1,2,3#^, Rongqin Zhang^3,4^, Taixuan Wan^1,2,3^, Xiaolin Pang^2,3,5,6*^, Zhanwen Zhang^3,4*^, Liang Huang^1,2,3*^*  ^1^Department of General Surgery (Colorectal Surgery), The Sixth Affiliated Hospital, Sun Yat-sen University, Guangzhou, China.  ^2^Guangdong Provincial Key Laboratory of Colorectal and Pelvic Floor Diseases, The Sixth Affiliated Hospital, Sun Yat-sen University, Guangzhou, China.  ^3^Biomedical Innovation Center, The Sixth Affiliated Hospital, Sun Yat-sen University, Guangzhou, China.  ^4^Department of Nuclear Medicine, The Sixth Affiliated Hospital, Sun Yat-sen University, Guangzhou, China  ^5^Department of Radiation Oncology, The Sixth Affiliated Hospital, Sun Yat-sen University, Guangzhou, China.  ^6^Department of General Surgery, The Sixth Affiliated Hospital, Sun Yat-sen University, Guangzhou, China.  ^#^ These authors contributed equally to this work.    **^*^Correspondence:**  **Xiaolin Pang (MD, PhD)**  Department of Radiation Oncology, Department of General Surgery, Guangdong Provincial Key Laboratory of Colorectal and Pelvic Floor Diseases, and Biomedical Innovation Center, The Sixth Affiliated Hospital, Sun Yat-sen University, Guangzhou, Guangdong 510655, China.  Email: [pangxl5@mail.sysu.edu.cn](mailto:pangxl5@mail.sysu.edu.cn)  **Zhanwen Zhang (MD, PhD)**  Department of Nuclear Medicine, and Biomedical Innovation Center, The Sixth Affiliated Hospital, Sun Yat-sen University, Guangzhou, Guangdong 510655, China.  Email: [zhzhanw7@mail.sysu.edu.cn](mailto:zhzhanw7@mail.sysu.edu.cn)  **Liang Huang (MD, PhD)**  Department of General Surgery (Colorectal Surgery), Guangdong Provincial Key Laboratory of Colorectal and Pelvic Floor Diseases, and Biomedical Innovation Center, The Sixth Affiliated Hospital, Sun Yat-sen University, Guangzhou, Guangdong 510655, China.  Phone: +862038455369; Fax: +862038254221; Email: [huangl75@mail.sysu.edu.cn](mailto:huangl75@mail.sysu.edu.cn)  **Supplementary Table 1. Quantitative ^18^F-FDG PET Parameters at Baseline and pre-operative, Stratified by pCR.** | | | | |  |
| --- | --- | --- | --- | --- | --- |
| Parameter | Subparameter | non-pCR | pCR | *P* |  |
| Baseline ^18^F-FDG | SUVmax (g/mL) | 10.5 (4.3) | 12.5 (5.4) | 0.352^b^ |  |
|  | SUVmean (g/mL) | 5.6 (1.8) | 6.7 (2.4) | 0.077^a^ |  |
|  | MTV (cm³) | 19.8 (9.9) | 17.7 (11.0) | 0.312^b^ |  |
|  | TLG (g/mL × cm³) | 112.1 (73.1) | 118.3 (103.3) | 0.898^b^ |  |
|  | SUVpeak (g/mL) | 8.4 (3.1) | 9.7 (4.0) | 0.441^b^ |  |
|  | TLR (%) | 6.9 (3.2) | 8.4 (3.6) | 0.143^b^ |  |
|  | TIMR (%) | 23.9 (13.2) | 28.5 (11.6) | 0.076^b^ |  |
| Preoperative ^18^F-FDG | SUVmax (g/mL) | 4.0 (1.4) | 4.1 (2.0) | 0.825^b^ |  |
|  | SUVmean (g/mL) | 2.7 (0.5) | 2.6 (0.6) | 0.694^a^ |  |
|  | MTV (cm³) | 6.4 (7.5) | 1.9 (2.3) | **0.043^b^** |  |
|  | TLG (g/mL × cm³) | 56.8 (164.4) | 7.1 (6.4) | **0.027^b^** |  |
|  | SUVpeak (g/mL) | 3.6 (0.8) | 3.6 (0.7) | NA |  |
|  | TLR (%) | 2.4 (0.8) | 2.5 (1.2) | 0.664^a^ |  |
|  | TIMR (%) | 7.8 (2.9) | 8.3 (4.0) | 0.769^b^ |  |
| Δ baseline to preoperative ^18^F-FDG | ΔSUVmax% | -54.9 (30.8) | -62.5 (22.9) | 0.295^b^ |  |
|  | ΔSUVmean% | -48.6 (16.7) | -56.5 (19.0) | 0.231^a^ |  |
|  | ΔMTV% | -73.7 (23.2) | -87.5 (15.1) | 0.057^b^ |  |
|  | ΔTLG% | -70.9 (59.4) | -91.9 (8.3) | **0.020**^b^ |  |
|  | ΔSUVpeak% | -54.4 (16.9) | -57.5 (23.1) | NA |  |
|  | ΔTLR% | -58.2 (30.2) | -65.7 (22.1) | 0.200^b^ |  |
|  | ΔTIMR% | -60.2 (24.4) | -66.0 (21.6) | 0.310^b^ |  |
| TBR = Tumor SUVmax / background SUVmean | | | | |  |
| Qualitative data are presented as n (%), continuous data are presented as mean (SD). | | | | |  |
| ^a^ Welch's *t*-test for comparison between non-pCR and pCR. | | | | |  |
| ^b^ Mann–Whitney *U* test for comparison between non-pCR and pCR. | | | | |  |
| Missing *P* values: For the parameters “preoperative ¹⁸F-FDG SUVpeak” and “ΔSUVpeak%”, a valid *P* value could not be calculated because one comparison group contained only 2 subjects, which precludes reliable statistical testing. These are designated as “NA” (not applicable) in the table. | | | | |  |
|  | | | | |  |
|  |  |  |  |  |  |
|  |  |  |  |  |  |

| **Supplementary Table 2. Performance of Individual Variables in Predicting pCR.** | | | | | | | |
| --- | --- | --- | --- | --- | --- | --- | --- |
| Parameter | Subparameter | Cutoff | AUC | 95% CI | *P* | Sensitivity (%) | Specificity (%) |
| Serum (Preoperative) | Preoperative IL-6 | 2.5 | 0.69 | 0.52 - 0.87 | 0.028 | 88.24% | 50.00% |
| Pathology | TLS | 1.5 | 0.72 | 0.57 - 0.87 | 0.004 | 71.43% | 63.64% |
|  | iTLS | 0.5 | 0.69 | 0.54 - 0.85 | 0.013 | 76.19% | 54.55% |
|  | Agg | 0.5 | 0.76 | 0.61 - 0.91 | 0.001 | 76.19% | 81.82% |
|  | B cells | 956.4 | 0.71 | 0.55 - 0.88 | 0.01 | 57.14% | 84.21% |
| ^18^F-FDG PET/CT Imaging | Preoperative ^18^F-FDG MTV | 4.1 | 0.69 | 0.52 - 0.85 | 0.027 | 89.47% | 45.45% |
|  | Preoperative ^18^F-FDG TLG | 21.3 | 0.73 | 0.52 - 0.85 | 0.010 | 100.00% | 38.89% |
| ^18^F-FAPI PET/CT Imaging | Preoperative ^18^F-FAPI FTV (T3) | 4.6 | 0.7 | 0.55 - 0.91 | 0.041 | 92.86% | 46.67% |
|  | Δ^18^F-FAPI TIMR (%) | 54.4 | 0.67 | 0.50 - 0.90 | 0.034 | 65.22% | 66.67% |
|  | RI_24_ 18F-FAPI TIMR | 29.3 | 0.71 | 0.51 - 0.83 | 0.020 | 81.25% | 60.00% |
|  | RI_34_ ^18^F-FAPI TIMR | 4.2 | 0.71 | 0.53 - 0.89 | 0.023 | 75.00% | 75.00% |
| Values represent optimal ROC cutoff, AUC (95% CI), sensitivity, specificity, and the P value for AUC >0.5. | | | | | | | |
| Abbreviations: pCR, pathologic complete response; AUC, area under the curve; CI, confidence interval; IL-6, interleukin-6; TLS, tertiary lymphoid structure; iTLS, intratumoral TLS; Agg, immune cell aggregates; α-SMA, alpha-smooth muscle actin; MTV, metabolic tumor volume; TLG, total lesion glycolysis; FAPI, fibroblast activation protein inhibitor; TIMR, tumor-to-iliacus ratio; Δ, percentage change from baseline; RI24/RI34. | | | | | | | |

| **Supplementary Table S3. Coefficients and odds ratios of the final integrative logistic regression model for pCR prediction.** | | | |
| --- | --- | --- | --- |
| Predictor | β coefficient | OR (95% CI) | Scaling |
| Preoperative ¹⁸F-FAPI TIMR(T1) | -1.082 | 0.339 (0.102–0.833) | per 1-unit increase |
| B-cell density | -3.515 | 0.030 (0.002–0.221) | per 100 cells/mm² increase |
| iTLS count | 1.556 | 4.741 (1.816–19.106) | per 1-unit increase |
| RI34 ¹⁸F-FAPI TIMR | -2.131 | 0.119 (0.007–0.946) | per 1-unit increase |
| Continuous candidate predictors were z-standardized for LASSO feature selection. The final logistic regression model was refitted using the selected predictors on the clinically interpretable scales shown in the “Scaling” column. B-cell density was entered as B-cell density/100; thus, its β coefficient and OR correspond to a 100 cells/mm² increase, whereas those of the other predictors correspond to a 1-unit increase. The reported ORs are not expressed per 1-standard-deviation increase. | | | |
| Abbreviations: β, regression coefficient; OR, odds ratio; CI, confidence interval; pCR, pathological complete response; FAPI, fibroblast activation protein inhibitor; TIMR, tumor-to-iliacus muscle ratio; iTLS, intratumoral TLS; RI34, retention index between the third and fourth imaging time points. | | | |

| **Supplementary Table S4. Leave-one-variable-out analysis of the final predictive model** | | | | | | |
| --- | --- | --- | --- | --- | --- | --- |
| Model | Predictors included | Mean AUC | 95% CI | Sensitivity | Specificity | ΔAUC vs full model |
| Full model | TIMR(T1), B-cell density, iTLS, RI_34_ TIMR | 0.874 | 0.858–0.890 | 75.10% | 74.40% | Reference |
| Without TIMR(T1) | B-cell density, iTLS, RI_34_ TIMR | 0.766 | 0.746-0.787 | 74.93% | 65.33% | -0.108 |
| Without B-cell density | TIMR(T1), iTLS, RI_34_ TIMR | 0.760 | 0.739-0.781 | 82.97% | 58.77% | -0.114 |
| Without iTLS | TIMR(T1), B-cell density, RI_34_ TIMR | 0.778 | 0.757-0.798 | 79.57% | 64.50% | -0.096 |
| Without RI34 TIMR | TIMR(T1), B-cell density, iTLS | 0.801 | 0.781-0.822 | 69.73% | 74.50% | -0.073 |
| AUC, sensitivity, and specificity were estimated using the same repeated cross-validation framework for all models. Each reduced model was refitted after removing the indicated predictor. ΔAUC was calculated as the mean AUC of the reduced model minus that of the full model. Classification thresholds were determined within the training data and applied to the corresponding held-out validation data. | | | | | | |
| The reported interval represents the 2.5th and 97.5th percentiles of the AUC distribution across repeated cross-validation runs and should not be interpreted as a conventional independent-sample confidence interval. | | | | | | |

| **Supplementary Table S5. Diagnostic performance of conventional expert-interpreted restaging MRI for identifying pathological complete response** | | | |
| --- | --- | --- | --- |
| MRI assessment | Pathological pCR | Pathological non-pCR | Total |
| MRI-predicted complete response | 2 | 1 | 3 |
| MRI-predicted non-complete response | 18 | 20 | 38 |
| Total | 20 | 21 | 41 |
| Diagnostic performance: sensitivity, 10.0%; specificity, 95.2%; PPV, 66.7%; NPV, 52.6%; accuracy, 53.7%. | | | |

**Supplementary Figure S1. Histopathologic support for the association between CAF marker expression and pretreatment ¹⁸F-FAPI uptake in rectal cancer.**

(A) Representative immunohistochemical staining of the CAF-related markers α-SMA and FAP-α in pretreatment rectal cancer biopsy specimens, illustrating different expression intensities quantified by H-score. Corresponding pretreatment ¹⁸F-FAPI PET SUVmax values are shown for each example, suggesting that tumors with stronger CAF marker expression tended to show higher FAPI uptake. Scale bars: 100 μm.

(B, C) Scatter plots showing the correlations between pretreatment ¹⁸F-FAPI SUVmax and the H-scores of α-SMA (B) and FAP-α (C). Modest but statistically significant positive correlations were observed for both markers (α-SMA: r = 0.335, P = 0.032; FAP-α: r = 0.359, P = 0.021), providing histopathologic support for an association between tumor FAPI uptake and CAF-related stromal activation (n = 43).

Statistical significance was determined using Pearson correlation analysis. Abbreviations: CAF, cancer-associated fibroblast; α-SMA, alpha-smooth muscle actin; FAP-α, fibroblast activation protein-alpha; H-score, histochemistry score; SUVmax, maximum standardized uptake value; PET, positron emission tomography.

**Supplementary Figure S2. TGFβ blockade enhances B-cell infiltration and TLS formation in CT26 tumors.**

(A) Representative multiplex immunofluorescence images of endpoint CT26 tumors from the indicated treatment groups, showing enhanced TLS organization, particularly in mice receiving 5-FU, anti-PD-1, and 1D11. Sections were stained for CD4, CD79A, CD3, and CD138, with DAPI as a nuclear counterstain. Scale bar, 100 μm.

(B) Quantification of CD79A⁺ cells per field in the indicated treatment groups.

(C) Quantification of the total number of TLS per tumor section in the indicated treatment groups.

**Supplementary Figure S3. Representative multiplex immunofluorescence images showing TLS organization and maturation in endpoint CT26 tumors from the indicated treatment groups.**

Representative multiplex immunofluorescence images of endpoint CT26 tumors from the indicated treatment groups, showing increased TLS organization and more prominent BCL6⁺ germinal-center-containing mature TLS following TGFβ blockade, particularly in mice receiving 5-FU, anti-PD-1, and 1D11. Sections were stained for CD4, CD79A, Ki67, and BCL6, with DAPI as a nuclear counterstain. Scale bar, 100 μm.
